# A Characteristic Interval Modeling Method for Simultaneous Detection of Multiple Metal Ions

**DOI:** 10.3389/fchem.2022.839633

**Published:** 2022-02-10

**Authors:** Feng-Bo Zhou, Chang-Geng Li, Hong-Qiu Zhu

**Affiliations:** ^1^ School of Information Engineering, Shaoyang University, Shaoyang, China; ^2^ School of Physics and Electronics, Central South University, Changsha, China; ^3^ School of Automation, Central South University, Changsha, China

**Keywords:** zinc smelting wastewater, partial least squares, ultraviolet visible spectrophotometry, characteristic interval modeling, multiple metal ions

## Abstract

Abstract Aiming at the problems of low accuracy and large prediction errors caused by the serious overlap of multi-metal spectral signals in zinc smelting industrial wastewater, a characteristic interval modeling method is proposed. First, according to the absorption spectra of mixed solution, the characteristic intervals of copper and nickel are preliminarily screened by using different partition lengths. Second, take the smallest root mean squares error of cross validation and the largest correlation coefficient as the evaluation indicators, compare the full-spectral model and each local model, and select the optimal feature sub-intervals of copper and nickel. Last, the partial least squares method is used to model the combined wavelengths of the optimal sub-intervals to realize the simultaneous detection of copper and nickel. The linear determination ranges are 0.3–3.0 mg/L for copper and nickel. the correlation coefficients of copper and nickel are 0.9974 and 0.9966, respectively. The results show that the method reduces the complexity of the wavelength variable screening process, improves the accuracy of the model, and lays the foundation for the accurate analysis of polymetallic ions in zinc smelting industrial wastewater.

## Introduction

The zinc hydrometallurgy is mainly composed of a series of processes such as roasting, acid leaching, electrowinning and casting (Yadav and Banerjee, 2018). When the production process is completed, a large number of industrial wastewater will be discharged, and a variety of metal ions will remain in the industrial wastewater (Zhou et al., 2019). The copper (Cu) and nickel (Ni) are two main ions in industrial wastewater (Zhang et al., 2018; Li et al., 2018). The random discharge of these ions will seriously pollute the environment (Luo et al., 2019). In zinc smelting wastewater treatment process, the content of each metal ion needs to be obtained first, and then removed by chemical means according to its content (Zahmatkesh, et al., 2017; Molognoni et al., 2018). At present, zinc production company mainly rely on manual detection of metal ion concentration in the laboratory (Kočanová et al., 2017), which makes the information lag in the production process and lack of basis for optimization and adjustment (Chen et al., 2017; Chen et al., 2018). Therefore, modern analysis methods are urgently needed for the rapid determination of polymetallic ions in wastewater (Dong et al., 2018).

The effective metal ion detection methods include ultraviolet visible (UV-Vis) spectrophotometry, inductively coupled plasma mass spectrometry, liquid chromatography and polarography (He et al., 2016; Li et al., 2017). Considering the characteristics that the ion concentration of zinc industrial wastewater reaches the trace level and the industrial production parameters need to be corrected in time through rapid determination (Walash et al., 2009), this paper chooses ultraviolet-visible spectrophotometry as the metal ion detection method, mainly because the ultraviolet-visible spectrophotometry has the following advantages: 1) this method has high sensitivity (10^−4^–10^−6^/L), which is in line with the concentration of ions in zinc hydrometallurgy industrial wastewater (Zhou et al., 2009; Ji et al., 2012). 2) Because this method calculates the substance concentration by substance absorbance, it meets the requirements of nondestructive determination of metal ion solution (Liu et al., 2017). 3) The instrument has the advantages of simple structure, low maintenance cost and low repetition rate, and is suitable for on-line analysis (Attimarad 2012; Merás et al., 2002). However, due to the coexistence of multiple heavy metal ions in the same period in zinc smelting industrial wastewater, their chemical characteristics are similar, their mutual influence is serious (Rastogi et al., 2015), their absorption spectra overlap seriously (Lambert et al., 2017), and the full spectra information contains a lot of redundant non characteristic information (Fedenko et al., 2017), resulting in low accuracy and large prediction error of ultraviolet visible spectral analysis model (Lepane et al., 2015; Lee et al., 2017). Therefore, the traditional multivariate linear analysis method based on full band information modeling is difficult to accurately detect the concentration of polymetallic ions in zinc smelting wastewater (Rote and Bari, 2009; Khan et al., 2020).

At present, the commonly used wavelength variable selection methods mainly include: interval partial least squares method, Monte Carlo uninformative variable elimination method and competitive adaptive weighting method. However, in the case of serious spectral overlap, the first method rely on experience to define the size of the wavelength division and the number of combination intervals, and the screening process often results in multiple selections or omissions of the characteristic wavelength variables. The latter two methods separately sample and screen each wavelength, the screening process is complex, and the sampling process is random, which will lead to inaccurate calculation of variable stability index, thus affecting the screening results of characteristic wavelength variables. Therefore, this paper proposes an efficient and high-precision characteristic interval modeling method. This method aims to select the optimal characteristic points of each ion to be measured, reduce the blank information, solve the problems of serious spectral masking and the difference of inhibition degree, and improve the model detection accuracy.

## Theory

The characteristic interval modeling method is a method for screening the characteristic wavelength variables with high contribution to the model to improve the accuracy of the model. This method performs overall and regional screening of the characteristic wavebands of each metal ion to be measured, avoids the instability of the random sampling process of a single wavelength, and reduces the complexity of the wavelength variable screening process. At the same time, through the evaluation index of local interval, the bands with serious spectral overlap of metal ions are effectively avoided, and the characteristic bands of metal ions are selected. The measurement indicators of characteristic interval variables are root mean squares error of cross validation (RMSECV) and correlation coefficient (*R* ^2^), and the two calculation formulas are as follows:

$$RMSECV=\sqrt{\frac{\sum_{i=1}^{n}\left({\hat{y}}_{i}-y_{i}\right)}{n-1}}$$ (1)

$$R^{2}=\frac{\sum_{i=1}^{n}{\left({\hat{y}}_{i}-\bar{y}\right)}^{2}}{\sum_{i=1}^{n}{\left(y_{i}-\bar{y}\right)}^{2}}$$ (2)

**FIGURE 1:**
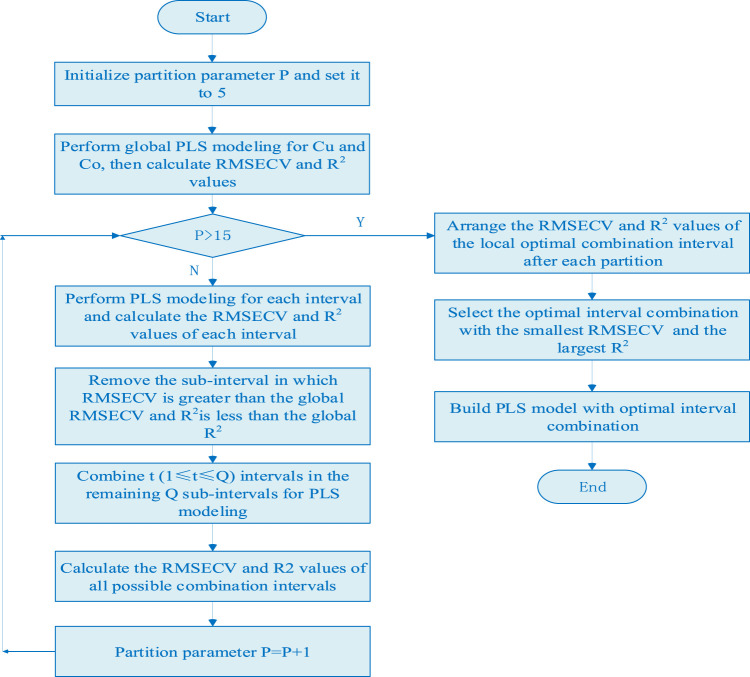
The flow chart of characteristic interval modeling method.

In Eqs 1, 2, $y_{i}$ is the actual value of the *i*th sample, ${\hat{y}}_{i}$ is the predicted value of the *i*th sample, $\bar{y}$ is the average value of the sample, and n is the number of samples in the calibration set. The smaller the RMSECV and the closer *R* ^2^ is to 1, the higher the accuracy of the model. Therefore, by comparing the RMSECV and *R* ^2^ values of each sub interval of each metal ion to be measured, the optimal sub interval set is selected. The basic steps of the algorithm are:

(1) The global PLS model of Cu and Ni are established in the full spectral range, and the RMSECV and *R* ^2^ values of Cu and Ni are calculated according to Eqs 1, 2 as the threshold of characteristic interval screening.
(2) Set P (5 < P < 15) cycles, divide the full spectra into P equal width sub intervals, establish the local PLS model of copper and nickel on each sub-interval, and calculate the RMSECV and *R* ^2^ values of copper and nickel in P sub-interval.
(3) Compare the RMSECV and *R* ^2^ values of the full spectral model and each local model, remove the wavelength interval in which RMSECV is greater than the global RMSECV and *R* ^2^ is less than the global *R* ^2^, and take out the remaining Q sub-interval with smaller RMSECV and larger *R* ^2^ value.
(4) Combine t (1 ≤ t ≤ Q) intervals in the remaining Q sub-intervals for PLS modeling, and calculate the RMSECV and *R* ^2^ values of all possible combination intervals.
(5) Similarly, taking the values of RMSECV and *R* ^2^ as the evaluation criteria of each combination interval, the interval combination corresponding to the minimum RMSECV and the maximum *R* ^2^ are selected as the optimal interval combination. The algorithm flow chart is shown in Figure 1.

## Experimental

### Apparatus and Reagents

The absorbance spectra were measured by a Purkinje T9 dual beam spectrophotometer (Beijing Purkinje General Instrument Ltd., China). The T9 spectrophotometer is a high-sensitivity scientific grade UV-Vis spectrophotometer, equipped with a high-performance PMT receiver and a special grating, mixed C-T dual monochromator and dual beam, so it has a high dynamic range and good SNR. The hexadecyl trimethyl ammonium bromide was used as stabilizer solution, nitroso R salt solution (0.4%) was chosen as chromogenic reagent, acetic acid-sodium acetate buffer (pH = 5.5) was prepared as buffer solution, and standard stock solutions of copper (12.5 mg/L) and nickel (12.5 mg/L) were prepared.

### Procedures

First, transfer the appropriate amount of copper and nickel standard solutions to a 25 ml volumetric flask. Then, add 2 ml nitroso R salt, 3 ml hexadecyl trimethyl ammonium bromide and 5 ml acetic acid-sodium acetate buffer. Finally, dilute and mix the solution with distilled water to ensure uniformity. The ultraviolet visible absorption spectra of the solution is measured with a reagent blank in the wavelength range of 350–600 nm. The prepared copper and nickel concentration ranges are both 0.3–3 mg/L 60 groups of copper and nickel mixed solutions in different proportions were prepared for spectral modeling analysis.

## Results and Discussion

### Spectral Characteristics

**FIGURE 2:**
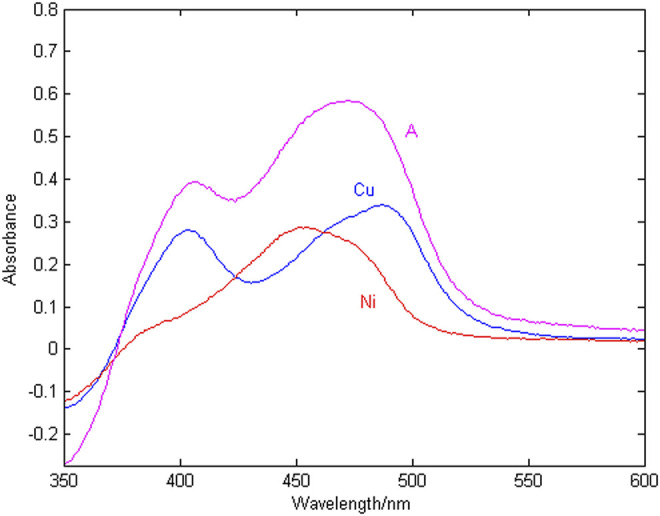
Absorption spectra of Cu, Ni and their mixture (A).

The spectral signal of copper and nickel single ion solution and the mixed solution are shown in Figure 2, where the copper concentration is 1.2 mg/L, the nickel concentration is 1.2 mg/L, and the wavelength detection range is 350–600 nm.

According to Figure 2, at the same concentration of 1.2 mg/L, the sensitivity of copper is higher than that of nickel. The peak of copper is at 405 and 488 nm, the peak of nickel is at 452 nm, copper and nickel overlap severely in the entire wavelength range. The reason is that copper and nickel ions are close neighbors in the periodic table, and their electrochemical characteristics are very similar, which makes the detection spectral signals overlap seriously, resulting in low spectral signal resolution and few characteristic wavelength variables. It is difficult to simultaneously detect the concentration of copper and nickel in zinc wastewater solution. Therefore, it is very important to select the characteristic wavelength to improve the resolution of copper and nickel absorption spectra.

### Univariate Calibration

**FIGURE 3:**
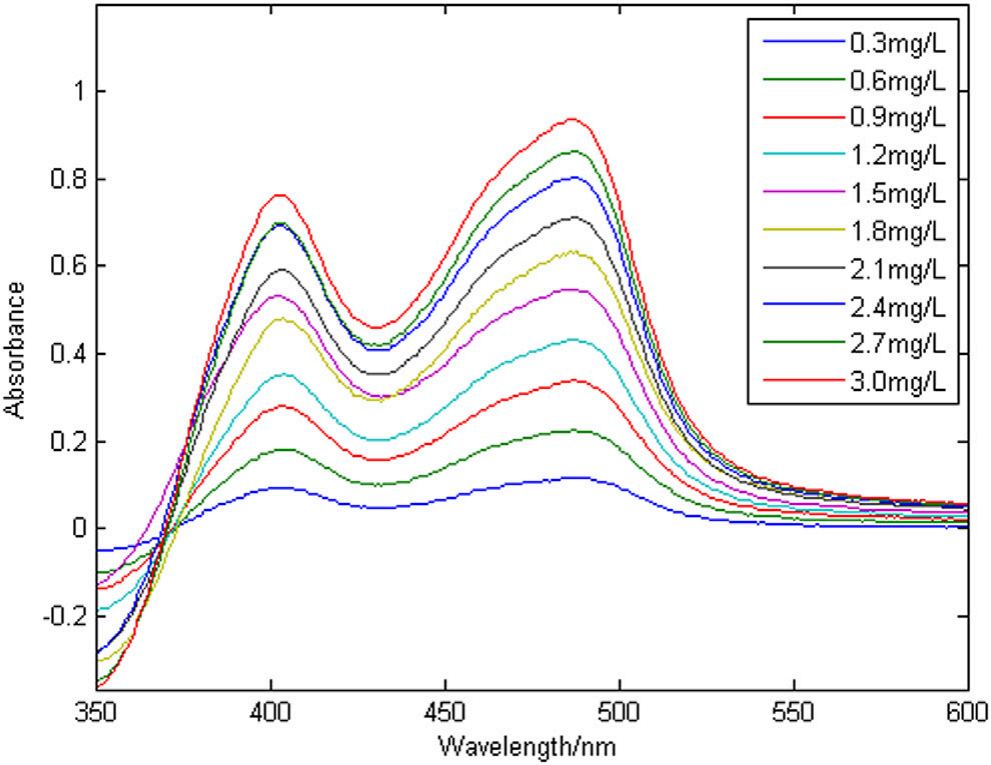
A group of absorption spectral signals of copper.

**FIGURE 4:**
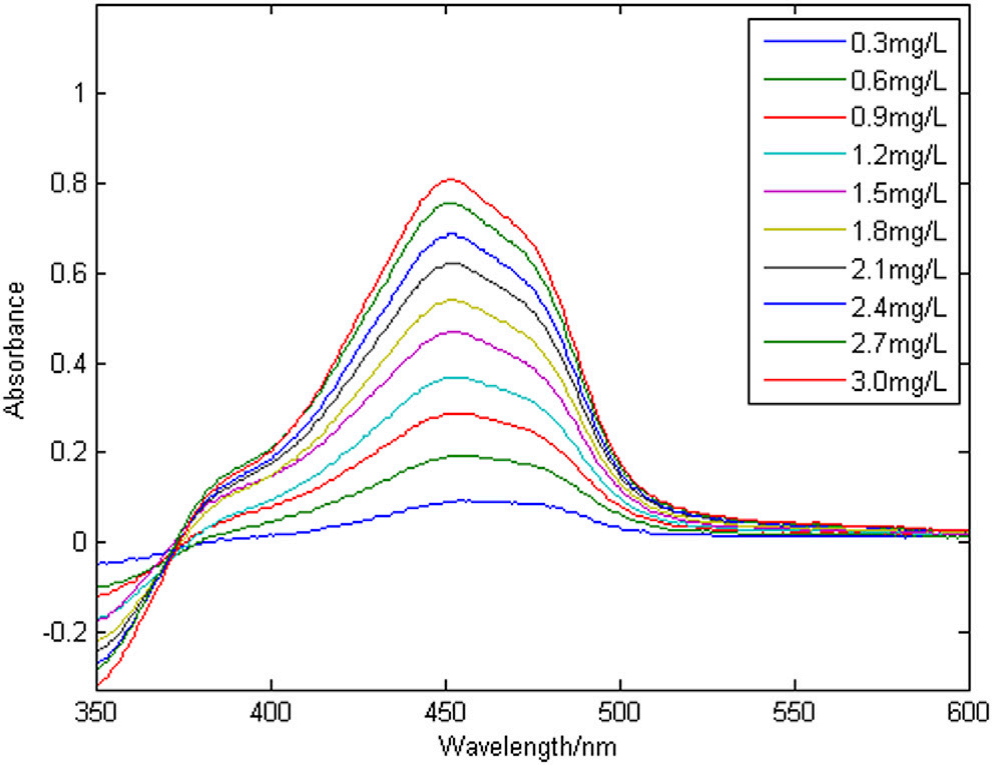
A group of absorption spectral signals of nickel.

Figure 3 shows a group of absorption spectra of Cu in zinc wastewater solution, in which the concentration range of Cu is 0.3–3 mg/L. Figure 4 shows a group of absorption spectra of Ni in zinc wastewater solution, in which the concentration range of Ni is 0.3–3 mg/L.

To evaluate the linearity of copper and nickel in the zinc wastewater solution, multiple wavelengths were randomly selected to perform linear fitting of absorbance and concentration. The linear regression equation and correlation coefficient (*R* ^2^) are shown in Table 1.

**TABLE 1:** The linearity evaluation index of copper and nickel.

Wavelength/nm	Linear regression equation		*R* ^2^	
	Cu	Ni	Cu	Ni
375	Y = 0.0164 X−0.0017	Y = 0.0038 X −0.0067	0.5089	0.4963
417	Y = 0.0696 X + 0.0143	Y = 0.0423 X + 0.0047	0.9662	0.9830
450	Y = 0.0723 X −0.0095	Y = 0.0890 X + 0.0122	0.9893	0.9934
496	Y = 0.1007 X + 0.0196	Y = 0.0268 X + 0.0114	0.9915	0.9842
517	Y = 0.0367 X + 0.0093	Y = 0.0072 X −0.0059	0.9802	0.9719
535	Y = 0.0169 X + 0.0037	Y = 0.0044 X + 0.0047	0.9601	0.9714

The correlation coefficient (*R* ^2^) is used to characterize the linear correlation of ions. The larger the value of *R* ^2^, the better the linearity. It can be seen from Table 1 that copper ion have no linearity at wavelengths of 375 nm. They are generally linear at 417 and 535 nm, and have good linearity at 450, 496 and 517 nm. The nickel ion has a small correlation coefficient (*R* ^2^) at wavelengths of 375 nm, so the linearity is very poor; it has good linearity at 417, 450 and 496 nm. Because the effective information of copper and nickel at different wavelength points is different, when modeling and detecting the concentration of copper and nickel by the partial least squares method, it is necessary to select the characteristic wavelength variables, and select the wavelength variables with high spectral signal sensitivity, large amount of information and strong linear correlation, so as to improve the accuracy of simultaneous detection of copper and nickel.

### Characteristic Interval Wavelength Selection

According to the steps of characteristic interval wavelength selection method described in section 2, the 30 sets of correction sets are modeled for the full-band wavelength variables of 350–600 nm, and the global RMSECV and *R* ^2^ values of copper are 0.9851 and 0.0674, and the global RMSECV and *R* ^2^ values of nickel are 0.9836 and 0.0712, which are used as the threshold for the subsequent screening of the characteristic interval. The whole band is divided into P sub intervals, PLS modeling is carried out respectively, and the RMSECV and *R* ^2^ values of P sub intervals are obtained. All sub intervals greater than RMSECV threshold and less than *R* ^2^ threshold are eliminated, and t (1 < t < Q) characteristic intervals are combined in turn in the remaining Q sub intervals to establish a joint interval PLS model. The combined modeling of the optimal characteristic intervals of copper and nickel after each interval division is shown in Table 2.

**TABLE 2:** The optimal interval combination of copper and nickel after each interval division.

Number of partitions	Optimal interval combination		RMSECV		*R* ^2^	
	Cu	Ni	Cu	Ni	Cu	Ni
5	[ 2 3 4]	[2 3]	0.0486	0.0499	0.9912	0.9943
6	[2 4 5]	[2 4]	0.0512	0.0518	0.9891	0.9866
7	[2 4 6]	[2 4 5]	0.0517	0.0520	0.9872	0.9857
8	[3 5 6]	[3 4 6]	0.0504	0.0488	0.9902	0.9952
9	[2 4 7]	[3 5 7]	0.0477	0.0511	0.9965	0.9913
10	[2 5 7]	[3 4 6]	0.0403	0.0533	0.9978	0.9848
11	[3 6 8]	[4 5 8]	0.0482	0.0477	0.9961	0.9956
12	[4 5 9]	[4 6 7]	0.0503	0.0459	0.9913	0.9965
13	[3 6 7]	[4 7 8]	0.0497	0.0485	0.9957	0.9950
14	[4 7 11]	[4 5 10]	0.0511	0.0509	0.9896	0.9922
15	[4 6 10]	[4 8 11]	0.0504	0.0514	0.9904	0.9881

**FIGURE 5:**
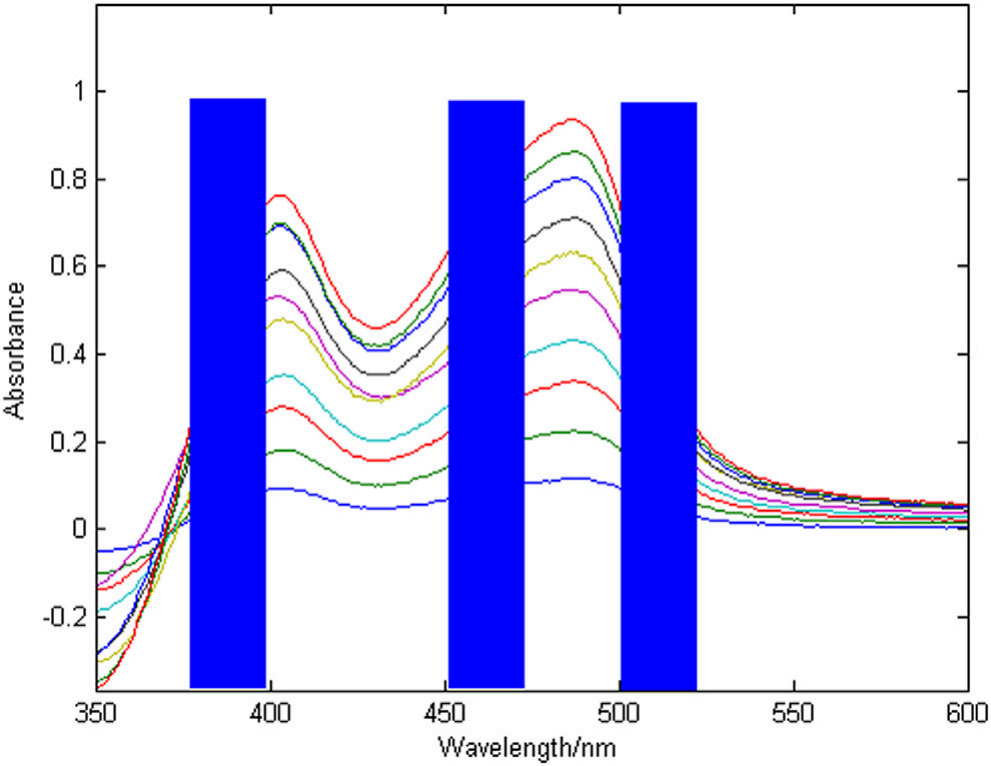
Combined wavelength screening results for copper characteristic intervals.

**FIGURE 6:**
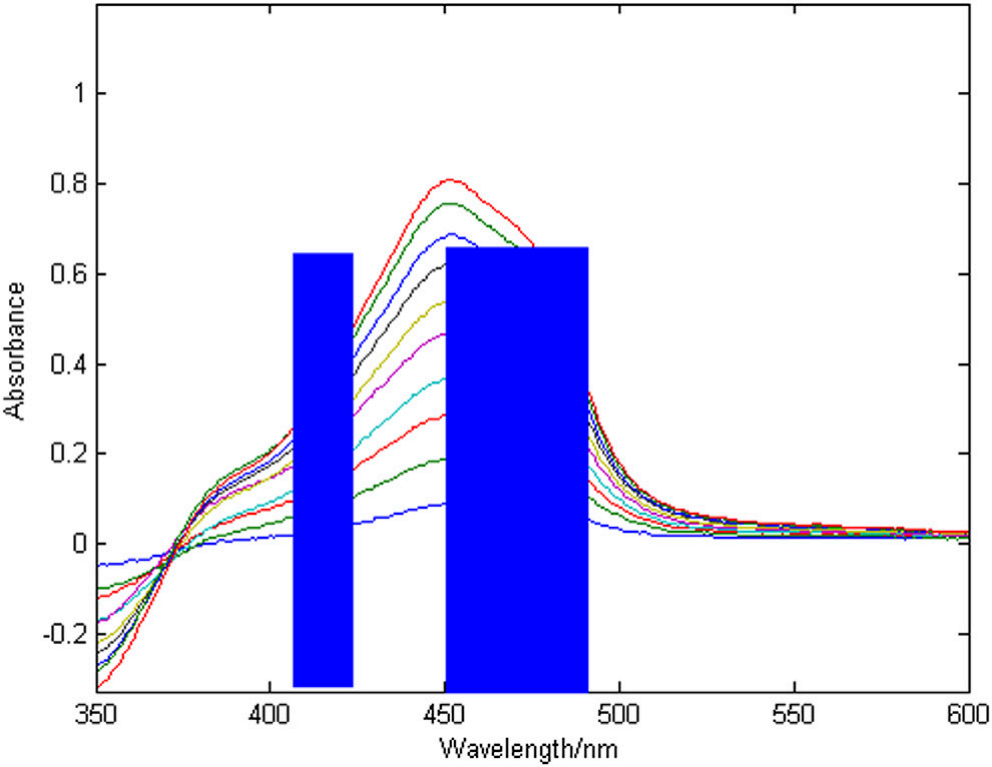
Combined wavelength screening results for nickel characteristic intervals.

It can be seen from Table 2 that the optimal characteristic interval combination of copper is to divide the whole band into 10 intervals, and when the second, fifth and seventh intervals are combined in sequence, the minimum value of RMSECV is 0.0403 and the maximum value of *R* ^2^ is 0.9978. The optimal feature interval combination of nickel is to divide the whole band into 12 intervals, and when the fourth, sixth and seventh intervals are combined in sequence, the minimum value of RMSECV is 0.0459 and the maximum value of *R* ^2^ is 0.9965. The results of the combined wavelength screening of characteristic intervals are shown in Figures 5, 6. The wavelength ranges corresponding to the optimal characteristic intervals of copper are 375–400 nm, 450–475 nm, and 500–525 nm. The wavelength range corresponding to the optimal characteristic intervals of nickel is 410–430 nm, 450–470 nm, and 470–490 nm.

### Performance Comparison of Different Algorithms

**FIGURE 7:**
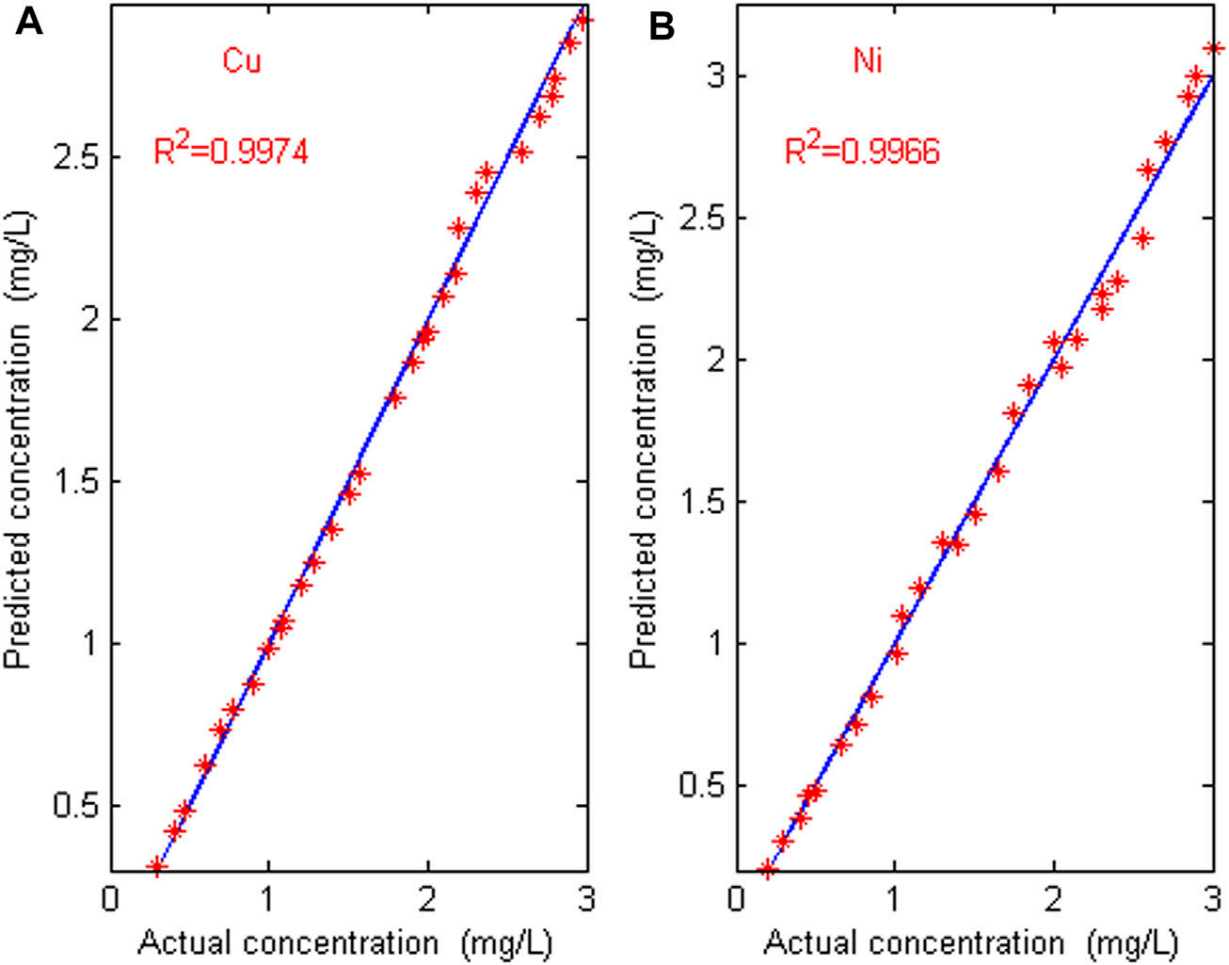
Error graph between predicted value and actual value. **(A)** The error graph of Cu; **(B)** the error graph of Ni.

The proposed characteristic interval modeling method (CIM) is used to detect the concentration of copper and nickel mixed solutions in 30 groups of prediction sets, and the performance is compared with full band partial least squares method (FBPLS), competitive adaptive weighting method (CARS) and Monte Carlo uninformative variable elimination method (MC_UVE). Table 3 shows the modeling comparison of four feature extraction methods. Using the calibration model by CIM, the predicted and actual values of Cu and Ni in mixture solution are shown in Figure 7.

**TABLE 3:** The modeling comparison of four feature extraction methods.

Detect ion	Evaluation index	FBPLS	CARS	MC_UVE	CIM
Cu	Maximum relative error	78.67%	26.84%	10.85%	7.16%
	Average relative error	28.61%	8.73%	7.79%	3.27%
	Number of variables	251	58	125	75
	RMSEP	0.3723	0.1187	0.0845	0.0413
	*R* ^2^	0.8972	0.8988	0.9936	0.9974
Ni	Maximum relative error	95.12%	39.20%	12.75%	8.33%
	Average relative error	38.14%	10.22%	8.76%	4.12%
	Number of variables	251	47	106	60
	RMSEP	0.3862	0.1451	0.0933	0.0457
	*R* ^2^	0.8916	0.8962	0.9935	0.9966

It can be seen from Table 3 that there are a large number of blank, overlapping and suppressed wavelengths in the full band wavelength, which interfere with the accurate and efficient modeling of ions, so the accuracy is very low and the real-time performance is poor. The CARS method selects fewer wavelengths, which is not ideal for measuring copper and nickel in zinc wastewater solution. The MC_UVE method still retains many redundant wavelength points when selecting variables, which makes the accuracy of the model worse. The proposed characteristic interval modeling method selects the region with higher sensitivity and reduces the masking and suppression differences of other ions to be measured. The predicted root mean square error (RMSEP) is lower, and the correlation coefficients (*R* ^2^) is higher. The average relative errors of Cu and Ni in the 30 validation sets are 3.27% and 4.12%. The maximum error of Cu is 7.16%, and the maximum error of Ni is 8.33%. The relative error is within 5%, which meets the requirements of industrial detection. As can be seen from Figure 7 that the predicted value is almost consistent with the actual value. The predicted root mean square errors (RMSEP) for Cu and Ni are 0.0413 and 0.0457, and the correlation coefficients (*R* ^2^) of Cu and Ni are 0.9974 and 0.9966, respectively. The results indicate that the proposed characteristic interval modeling method can effectively realize the simultaneous detection of copper and nickel in zinc industrial wastewater.

## Conclusion

A variety of heavy metal ions coexist in zinc industrial wastewater. Their chemical properties are similar, their mutual influence is serious, and their absorption spectra overlap severely. The full spectra information contains a lot of redundant non-characteristic information, which leads to low accuracy of analysis models and large prediction errors. Therefore, the traditional multi-variable linear analysis method of modeling the full-band information is difficult to achieve accurate detection of polymetallic ions in zinc smelting wastewater. Aiming at the problems of low accuracy and large prediction errors caused by the serious overlap of multi-metal spectral signals in zinc smelting industrial wastewater, a characteristic interval modeling method is proposed. This method screens wavelength variables in a coordinated and partitioned manner, avoids overlapping serious bands, ensures the stability of the screening process, reduces the complexity of wavelength screening, and improves the accuracy of the model. The proposed method is successfully used to analyze the severely overlapping spectra of Cu and Ni. The results show that the characteristic interval modeling method has obvious advantages in the separation of overlapping spectra compared with other methods. The proposed method lays a solid foundation for the accurate analysis of polymetallic ions in zinc smelting industrial wastewater.

## Data Availability

The raw data supporting the conclusion of this article will be made available by the authors, without undue reservation.
